# Feasibility and User Experience of Virtual Reality Positive Psychological Intervention in Older Korean Immigrants

**DOI:** 10.1093/geroni/igaf122.1455

**Published:** 2025-12-31

**Authors:** Soonhyung Kwon

**Affiliations:** University of South Florida, Tampa, Florida, United States

## Abstract

Older Korean immigrants in the U.S. face significant mental health challenges, particularly discrimination-related stress, anxiety, and depression, exacerbated by anti-Asian racism during the COVID-19 pandemic. Limited access to culturally and linguistically appropriate mental health care necessitates innovative approaches, such as virtual reality (VR)-based positive psychological interventions. This study examined the feasibility, acceptability, and user experience of a culturally tailored VR-based positive psychological intervention for older Korean immigrants during the pandemic. A mixed-methods approach was employed. The three-week (four-module) intervention assessed feasibility through recruitment, retention, adherence, and dropout rates. Acceptability and user experience were evaluated using surveys and semi-structured interviews. Feasibility was supported by high retention (96.9%) and engagement. Participants reported positive emotional engagement, cultural relevance, and ease of use. Participants found the VR intervention engaging, particularly enjoying the immersive nature-based environments and interactive exercises, which fostered a sense of achievement, gratitude, and a positive mindset. However, some faced technical difficulties and physical discomfort, such as pressure from the headset and eye strain due to blurred screens. A VR-based culturally tailored positive psychological intervention is a feasible and acceptable tool for addressing mental health disparities in older Korean immigrants. Future research should refine usability and assess long-term effectiveness for broader implementation.

